# Identifying the predictors of adherence to oral endocrine therapy in racial/ethnic minority patients with low socioeconomic status

**DOI:** 10.21203/rs.3.rs-2379786/v1

**Published:** 2022-12-22

**Authors:** Sama Rahimi, Onyebuchi Ononogbu, Anjana Mohan, Daniel Moussa, Susan Abughosh, Meghana Trivedi

**Affiliations:** University of Houston College of Pharmacy; University of Houston College of Pharmacy; University of Houston College of Pharmacy; University of Houston College of Pharmacy; University of Houston College of Pharmacy; University of Houston College of Pharmacy

**Keywords:** Breast cancer, tamoxifen, aromatase inhibitor, medication non-adherence, COVID-19 pandemic, racial minority, ethnic minority

## Abstract

**Background:**

Adherence to oral endocrine therapy (OET) is crucial in ensuring its maximum benefit in prevention and treatment of hormone receptor-positive (HR+) breast cancer (BC) in patients. Medication use behavior is suboptimal especially in racial/ethnic minorities of lower socioeconomic status (SES). We aimed to assess the OET adherence and its predictors in racial/ethnic minority patients of lower SES.

**Aim:**

We aimed to assess the OET adherence and determine the predictors of OET nonadherence in racial/ethnic minority patients of lower SES.

**Method:**

A retrospective study was conducted at the Harris Health System in Houston, Texas. Since the study period included the COVID-19 pandemic, data was collected during the 6 months prior and 6 months after the start of the pandemic. The adherence was assessed using the prescription refill data using the proportion of days covered. Multivariable logistic regression model was used to identify predictors of nonadherence. Eighteen years or older patients on appropriate doses of OET for prevention or treatment of BC were included.

**Result:**

In 258 patients, the adherence was significantly lower during the pandemic (44%) compared to before the pandemic (57%). The predictors of OET nonadherence before the pandemic were Black/African American, obesity/extreme obesity, prevention setting, tamoxifen therapy, and 4 or more years on OET. During the pandemic, prevention setting and those not using home delivery were more likely to be nonadherent.

**Conclusion:**

Racial/ethnic minority patients of lower SES, especially African Americans and those using OET for prevention of BC, require individualized interventions to improve adherence.

## Introduction

Hormone receptor-positive (HR+) breast cancer (BC) is the most common molecular subtype found in about 80% of BC patients [1–3]. In HR + BC patients, better adherence to oral endocrine therapy (OET) results in superior outcomes [4–9]. Despite a 30–50% reduction in recurrence risk by 5–10 years of OET, nonadherence rate is high among the patients [10–12]. The Food and Drug Administration (FDA)-approved OETs used are tamoxifen and Aromatase Inhibitors (AIs) such as anastrozole, exemestane, and letrozole [13–18]. Nonadherence to OET is more common among racial/ethnic minority groups compared to non-Hispanic whites [19–24] and in patients with financial burdens [20, 22, 25]. Higher OET nonadherence in racial/ethnic minorities and low-income patients likely contributes to the disparity in their outcomes [26–28].

Cancer health disparity has been a significant societal burden in the United States (U.S.). High out-of-pocket medical cost and unemployment rates due to cancer were common causes for treatment refusal or nonadherence in cancer patients [29, 30]. The COVID-19 pandemic has further increased the stress level of already vulnerable cancer patients because of the fear of contracting the virus and inadequate access to and utilization of health care, especially in those with low SES [31–33]. Therefore, accessible and equitable cancer care has been markedly compromised in racial/ethnic minority cancer patients with low SES during the pandemic [34, 35]. The barriers to OET adherence in racial/ethnic minority patients with low SES are poorly understood.

## Aim

The goal of this study was to assess the OET adherence and determine the predictors of OET nonadherence in racial/ethnic minority patients of lower SES.

### Ethical approval

The study was approved by the institutional review board of University of Houston (STUDY00001818, approved on Jan 30,2020) and Harris Health System (Protocol 20-02-2286, approved on March 3, 2020) with a waiver of informed consent.

## Methods

### Study Design and Data Source

This retrospective, single-center study included HR + BC patients on OET at Harris Health System in Houston, Texas, which is a county hospital system serving minority patients of low SES. A list of patients of Harris Health System with at least one OET dispensed record from June 2019 through September 2020 was extracted from the EPIC Electronic Health Record (EHR) system. The dispense history from June 2019 through August 2019 was used to determine whether the patients had OET supply in and after September 2019.

### Data Management

We collected the following data: patient demographics, height and weight to calculate body mass index (BMI), chronic diseases and comorbidities, diagnoses date, pathological and clinical cancer stage at diagnosis, HR and HER2 status, OET name and dosing, date of first OET prescription and initiation date, OET frequency dispense data, OET dispense quantity, date of OET discontinuation, any change in OET therapy, reason for discontinuation or change, number of month’s supply that was covered using mail-order drug delivery after March 31st, 2020, and number of telemedicine appointments after March 31st, 2020. The patients’ prescription refill data included the combination of prescription dispensed data of any outpatient pharmacy with an integrated e-prescribing system. Thirty patients were included in piloted data collection to review any necessary update in the data collection form and crosscheck the consistency of data collection by two investigators. Queries were resolved by licensed and board-certified oncology pharmacists (MVT and OO). Random audits were performed during and after data collection to ensure data integrity.

### Study Population

Inclusion criteria included patients with at least 18 years of age, seen and followed at Harris Health System, of any stage of HR + BC, and those whose OET was not discontinued due to death or progression. Patients not taking appropriate doses for OET (tamoxifen 20 mg once daily, anastrozole 1 mg once daily, letrozole 2.5 mg once daily, and exemestane 25 mg once daily) or on OET for reasons other than HR + BC were excluded.

### Study Variables

The primary endpoint was OET adherence during the 6 months prior to (September 2019 through February 2020) and 6 months after the start of the COVID-19 pandemic (April 2020 through September 2020). Adherence was measured using the proportion of days covered (PDC), which was calculated by dividing the total number of prescription days covered during each period by the total of days in each period. Patients should have received at least 80% of a day’s supply within each 6 months period to qualify as adherent [36]. Therefore, in each period, at least 220 days’ supply of medication was required for the patients to be considered adherent. Independent variable selection was guided by the Andersen Behavioral Model for Healthcare Resource Use Behavior including predisposing, enabling and need factors [37]. Predisposing factors included age, race/ethnicity, BMI, type, of OET years on OET. Enabling factors included home delivery during COVID-19 and use of telehealth during COVID-19. Need factors included cancer stage, HER2, and comorbidities including metabolic syndrome and depression.

### Statistical Analysis

Adherence before and during the pandemic was compared using Student’s paired t-test or McNemar chi-square test when PDC was used as a continuous or categorical variable, respectively. Two multinomial regression models were conducted to identify the predictors of adherence to OET during each of the 6 months periods. The predictors included in the model were age, race/ethnicity, BMI, cancer stage, HER2, type of OET, years on OET, metabolic syndrome, and depression. Home delivery during COVID-19 and use of telehealth during COVID-19 were the additional predictors included in the model during the pandemic. All the statistical analysis was done using Statistical Analysis System (SAS) version 9.4 (SAS Institute, Cary, NC) at a priori significance level of 0.05.

## Results

Out of 270 patients, 12 patients were excluded for taking OET for reasons other than HR + BC. The study cohort included 258 patients.

### Baseline Characteristics

The baseline demographic and clinical characteristics of patients are presented in Table 1. These characteristics were representative of the patient population of Harris Health System. Among 258 patients that were included in the data analysis, 54 patients were diagnosed with non-invasive (stage 0) BC or ductal/lobular hyperplasia. Out of these 54 patients, 39 (72%) patients had ductal carcinoma in situ (DCIS), 6 (11%) patients had lobular carcinoma in situ (LOIS), 8 (15%) patients had ductal hyperplasia, 1 (2%) had lobular hyperplasia.

### Impact of COVID-19 pandemic on OET adherence

The mean PDC from September 2019 to February 2020 was 0.72 and from April 2020 to September 2020 was 0.52. Of the 258 patients, 112 (43%) patients were nonadherent before the start of COVID-19 pandemic and 171 (66%) patients were nonadherent during the pandemic. There was a significant difference in the adherence before and during the pandemic when PDC was used as a continuous (p < 0.0001, Student’s paired t-test) or a categorical variable (p < 0.0001, McNemar chi-square test).

### Predictors of nonadherence before the pandemic

A multivariate logistic regression analysis of data 6 months before the pandemic identified several factors significantly associated with OET non-adherence. Black/African American and White/Caucasian were less likely to be adherent compared to Hispanic/Latino (Black/African American: odds ratio (OR), 0.43; 95% confidence interval [CI], 0.22–0.84; White/Caucasian: OR, 0.20; 95% CI, 0.05–0.73). Obese or extremely obese patients (BMI of ≥30) were less likely to be adherent compared to those with BMI of < 30 (OR, 0.55; 95% CI, 0.30–1.01). Patients diagnosed with invasive BC (stages 1–4) were more likely to be adherent compared to those diagnosed with non-invasive tumors or ductal/lobular hyperplasia. Patients on aromatase inhibitors were more likely to be adherent compared to patients on tamoxifen (OR, 2.60; 95% CI, 1.26–5.36). Patients taking OET for four years or longer were less likely to be adherent compared to those who were on OET for less than four years (OR, 0.29; 95% CI, 0.13–0.65) (Table 2).

### Predictors of nonadherence during the pandemic

Patients who were diagnosed with invasive BC (stages 1–3) were more likely to be adherent compared to those diagnosed with non-invasive tumors or ductal/lobular hyperplasia. Patients using home delivery at least once were more likely to be adherent compared to those not using home delivery (OR, 25.42; 95% CI, 7.44–86.81) (Table 3). The proportion of patients using home delivery was not significantly different between racial/ethnic groups.

## Discussion

### Statement of key findings

This was a retrospective, single-center study evaluating the OET adherence and predictors of nonadherence in racial/ethnic minority patients of low SES. We found that OET adherence was low and was further reduced during the pandemic in patients seen at the Harris Health System that serves the indigent population of Houston. This is the first study reporting the impact of the COVID-19 pandemic on OET adherence in HR + BC patients. Even before the pandemic, OET adherence was low at only 57% in patients with low SES. This was lower than OET adherence of 82% at the Houston Methodist Hospital serving insured patients in the same geographic location of Houston before the pandemic in our previous study [38]. The pandemic further and significantly reduced OET adherence to only 44% in Harris Health patients. COVID-19 has been found to negatively impact cancer outcomes in racial/ethnic minorities and patients of low SES [39–41]. While access to life-saving medications such as OET was provided to Harris Health patients by introducing home delivery, it was not utilized by all patients, which significantly impacted the adherence during the pandemic. Identifying prohibitive factors for using home delivery is necessary to optimize outcomes in this patient population.

### Strengths and Weaknesses

Strengths of our study include the significance of the research, novelty of our findings, and the study design. Harris Health System currently does not have an existing program to improve adherence. Findings of this study can be used to design and implement future interventions and programs customized to African Americans and Hispanic patients to improve their long-term adherence. Our study provides critical information for preparedness for future pandemic or natural disasters and for the future study conducting real-time assessment of adherence and implementation of intervention in ambulatory oncology clinic.

Some limitations of this study are described below. The study was conducted in one center, which can limit generalizability of findings to similar centers and geographic locations. The relatively small sample size may have impacted the power to detent significant differences and lead to wide confidence intervals with some variables. Refilling the medications does not guarantee the patient actually consumed the medication as prescribed. Additionally, residual uncontrolled confounding may exist as some variables that can impact adherence were not controlled for, like educational level, income, family support, and other comorbidities. Despite these limitations, the study has found the low adherence rate among racial/ethnic minorities of low SES, underscoring the need for tailored interventions to enhance adherence in this population.

### Interpretation

We found that patients at higher risk of invasive BC, namely those diagnosed in non-invasive BC or with ductal/lobular hyperplasia, were less likely to be adherent to OET. Most studies to date have evaluated adjuvant OET adherence in the setting of early-stage BC, not for prevention [19–24]. Our study emphasizes an important need to evaluate barriers to OET adherence in the prevention setting and for tailored interventions to improve OET adherence since it reduces the risk of new BC diagnosis.

Our results of lower OET adherence in Black/African Americans are consistent with published literature [23, 22, 20, 21, 24]. African Americans have a higher incidence of certain cancers and face greater hurdles to cancer prevention, detection, and treatment [42]. As a result, African Americans have the highest death rate and shortest survival of any racial/ethnic group for most cancers [42–44]. Despite slightly lower rates of breast cancer (BC) diagnosis, African American women are >40% more likely to die from it than Caucasians in Texas and the U.S. [42, 26, 45, 43, 27]. The most common barrier to OET adherence reported by patients and physicians is adverse drug reactions [46–48]. African American women more often report postmenopausal symptoms and joint pain with OET than Caucasians [49, 50]. They also more often believe that their recurrence risk would not change if they stopped OET and report forgetting to take it regularly [51, 49, 52, 50]. Therefore, patient-centered interventions addressing these key barriers to OET adherence in African American women, especially those of low SES, are urgently needed.

A large subset (62%) of patients in our study was obese or extremely obese, which was also associated with nonadherence. Higher body weight has been associated with worse BC-specific outcomes in patients, and body weight management is linked to favorable BC-specific survival [53, 54, 17, 55]. It is possible that patients who are more health-conscious in general are more likely to maintain ideal body weight and stay on therapy. Therefore, adequate patient education as well as interventions to improve overall health including weight management and OET adherence are necessary to improve overall BC outcomes in HR + BC patients with low SES.

### Further research

Our findings were communicated to the breast medical oncology and the cancer prevention team as well as the Pharmacy Department at the Harris Health Hospital. Department of Pharmacy had instituted the home delivery option during the pandemic. Since not using home delivery adversely affected OET adherence, encouraging patients to use it could help improve their OET adherence. The home delivery option is still available to all Harris Health patients, and its optimized use could lower the burden of the pandemic on patients with low SES. Future directions for research include developing patient-centered interventions and testing their feasibility and effectiveness to improve treatment outcomes and survival in racial and ethnic minorities of lower SES.

## Conclusion

In conclusion, OET adherence is a major concern in racial/ethnic minorities of low SES with HR + BC. The COVID-19 pandemic adversely affected OET adherence. Therefore, patient-centered interventions tailored to Black/African American patients and those using OET for prevention are urgently needed to improve BC outcomes in this population.

## Figures and Tables

**Table 1:** Baseline Characteristics (N = 258)

Variable	N (%)
**Age**	
Below 65 years	196 (76.0)
65 years and above	62 (24.0)
Race/Ethnicity	
Black/African American	66 (25.6)
Hispanic/Latino	159 (61.6)
White/Caucasian	14 (5.4)
Others	19 (7.4)
Body Mass Index	
< 30	98 (38.0)
≥ 30	160 (62.0)
Stage	
0 or Ductal/lobular hyperplasia	55 (21.3)
I	71 (27.5)
II	65 (25.2)
III	48 (18.6)
IV	19 (7.4)
HER2	
HER2-negative	214 (83.0)
HER2-positive	29 (11.2)
N/A	15 (5.8)
Oral endocrine therapy	
Tamoxifen	100 (38.8)
Aromatase Inhibitors	157 (60.8)
N/A	1 (0.4)
Years on oral endocrine therapy	
≤ 1 year	120 (46.5)
2–3 years	80 (31.0)
≥ 4 years	58 (22.5)
Metabolic syndrome	
No	72 (27.9)
Yes	186 (72.1)
Depression	
No	204 (79.1)
Yes	54 (20.9)
Home Delivery (during COVID-19 pandemic)	
0	87 (33.7)
≥ 1 home delivery	171 (66.3)
Telemedicine (during COVID-19 pandemic)	
0	76 (29.5)
≥ 1	182 (70.5)

**Table 2:** Predictors of Adherence to OET Before COVID-19 Pandemic

Variable	Adjusted OR	95% CI	P-value
**Age**			
Below 65 years	Ref		
65 years and above	1.90	0.91–3.96	0.08
Race/Ethnicity			
Hispanic/Latino	Ref		
Black/African American	0.43	0.22–0.84	0.01^a^
White/Caucasian	0.20	0.05–0.73	0.01^a^
Others	1.53	0.45–5.11	0.48
Body Mass Index			
< 30	Ref		
≥ 30	0.55	0.30–1.01	0.05^a^
Stage			
0 or Ductal/lobular hyperplasia	Ref		
I	13.34	4.71–37.73	< 0.0001^a^
II	6.74	2.52–17.99	0.0001^a^
III	7.45	2.55–21.74	0.0002^a^
IV	7.95	1.96–32.25	0.003^a^
HER2			
HER2-negative	Ref		
HER2-positive	0.85	0.33–2.16	0.73
Oral endocrine therapy			
Tamoxifen	Ref		
Aromatase Inhibitors	2.60	1.26–5.36	0.009^a^
Years on oral endocrine therapy			
≤ 1 year	Ref		
2–3 years	0.78	0.40–1.51	0.46
≥ 4 years	0.29	0.13–0.65	0.002^a^
Metabolic syndrome			
No	Ref		
Yes	1.11	0.56–2.18	0.75
Depression			
No	Ref		
Yes	0.76	0.36–1.58	0.46

Abbreviations: CI-Confidence interval; OET = Oral endocrine therapy; OR = Odds ratio.

A p-value ≤ 0.05 was considered statistically significant.

a Statistically significant

**Table 3:** Predictors of Adherence to OET During COVID-19 Pandemic

Variable	Adjusted OR	95% CI	P-value
**Age**			
Below 65 years	Ref		
65 years and above	0.766	0.34–1.70	0.51
Race/Ethnicity			
Hispanic/Latino	Ref		
Black/African American	1.10	0.51–2.34	0.80
White/Caucasian	0.45	0.11–1.85	0.27
Others	3.08	0.82–11.51	0.09
Body Mass Index			
< 30	Ref		
≥ 30	0.72	0.37–1.41	0.34
Stage			
0 or Ductal/lobular hyperplasia	Ref		
I	3.95	1.27–12.30	0.01^a^
II	3.54	1.15–10.90	0.02^a^
III	4.82	1.38–16.84	0.01^a^
IV	3.13	0.68–14.35	0.14
HER2			
HER2-negative	Ref		
HER2-positive	2.26	0.50-10.27	0.43
Oral endocrine therapy			
Tamoxifen	Ref		
Aromatase Inhibitors	1.14	0.54–2.39	0.72
Years on oral endocrine therapy			
≤ 1 year	Ref		
2–3 years	0.76	0.37–1.56	0.46
≥ 4 years	0.59	0.24–1.49	0.26
Metabolic syndrome			
No	Ref		
Yes	0.82	0.39–1.72	0.61
Depression			
No	Ref		
Yes	1.20	0.55–2.64	0.63
Home Delivery (during COVID-19 pandemic)			
0	Ref		
≥ 1 home delivery	25.42	7.44–86.81	<0.0001^a^
Telemedicine (during COVID-19 pandemic)			
0	Ref		
≥ 1	1.5	0.71–3.21	0.27

Abbreviations: CI-Confidence interval; OET = Oral endocrine therapy; OR = Odds ratio.

A p-value ≤ 0.05 was considered statistically significant.

a Statistically significant

## Impact Statements

- This is the first study reporting that the adherence to OET for breast cancer prevention or treatment was reduced in patients of low socioeconomic status during the COVID-19 pandemic.
- We have found that patients taking OET for prevention has worse adherence compared to those taking it for treatment of HR + BC; Black/African Americans had worse adherence than Hispanic patients.
- Patients who used home delivery had better adherence during the pandemic, suggesting that this intervention may be useful even during the non-pandemic times.
